# Autoimmune rheumatic disease IgG has differential effects upon neutrophil integrin activation that is modulated by the endothelium

**DOI:** 10.1038/s41598-018-37852-5

**Published:** 2019-02-04

**Authors:** Akif A. Khawaja, Charis Pericleous, Vera M. Ripoll, Joanna C. Porter, Ian P. Giles

**Affiliations:** 1 Centre for Inflammation and Tissue Repair, University College London, London, United Kingdom; 2 Centre for Rheumatology, University College London, London, United Kingdom; 3 National Heart and Lung Institute, Imperial College London, London, United Kingdom

## Abstract

The importance of neutrophils in the pathogenesis of autoimmune rheumatic diseases, such as systemic lupus erythematosus (SLE) and rheumatoid arthritis (RA), is increasingly recognised. Generation of reactive oxygen species (ROS) and release of neutrophil extracellular traps (NETs) by activated neutrophils are both thought to contribute to pathology; although the underlying mechanisms, particularly the effects of IgG autoantibodies upon neutrophil function, are not fully understood. Therefore, we determined whether purified IgG from patients with SLE or RA have differential effects upon neutrophil activation and function. We found that SLE- and RA-IgG both bound human neutrophils but differentially regulated neutrophil function. RA- and SLE-IgG both increased PMA-induced β_1_ integrin-mediated adhesion to fibronectin, whilst only SLE-IgG enhanced α_M_β_2_ integrin-mediated adhesion to fibrinogen. Interestingly, only SLE-IgG modulated neutrophil adhesion to endothelial cells. Both SLE- and RA-IgG increased ROS generation and DNA externalisation by unstimulated neutrophils. Only SLE-IgG however, drove DNA externalisation following neutrophil activation. Co-culture of neutrophils with resting endothelium prevented IgG-mediated increase of extracellular DNA, but this inhibition was overcome for SLE-IgG when the endothelium was stimulated with TNF-α. This differential pattern of neutrophil activation has implications for understanding SLE and RA pathogenesis and may highlight avenues for development of novel therapeutic strategies.

## Introduction

Systemic lupus erythematosus (SLE) and rheumatoid arthritis (RA) are both autoimmune rheumatic diseases (ARDs) that share features of endothelial dysfunction, aberrant leukocyte activation and pathogenic autoantibody formation, all of which contribute to pathophysiology. Increasingly, neutrophil dysfunction has also been recognised in ARD immunopathology^1,2^.

Neutrophils are rapidly recruited to sites of infection or inflammation, where complex interactions between neutrophil selectins and integrins with their endothelial ligand counterparts regulate neutrophil extravasation and activation. Activated neutrophils fight infection and promote inflammation via phagocytosis, degranulation and neutrophil extracellular trap (NET) formation. NET release results in the externalisation of a meshwork of chromatin fibres decorated with antimicrobial proteins and proteases, via a process termed NETosis^3^. NETs ensnare bacteria and promote pathogen killing, but also expose neo-antigens and pro-inflammatory molecules. Aberrant NETosis can induce endothelial damage and dysfunction^4,5^, leading to increased risk of atherosclerosis and vascular thrombosis^6,7^. Moreover, NETs have been demonstrated to activate both leukocytes^8–11^ and stromal cells^12^, which can drive disease progression. Dysregulation of this process has been implicated in both SLE and RA^13–15^.

Neutrophils express several different classes of integrins but the most important are the β_1_ and β_2_ integrins. The β_1_ integrins recognise ligands in the extracellular matrix (ECM), in particular those with the Arg-Gly-Asp (RGD) motif (found in fibronectin, vitronectin and laminin). As well as recognising RGD, α_4_β_1_ has a ligand-binding site for Leu-Asp-Val-Pro (LDVP) and Ile-Asp-Ala-Pro (IDAP) found in fibronectin. In addition, α_4_β_1_ can also bind the Ile-Asp-Ser-Pro (IDSP) motif found in vascular cell adhesion molecule (VCAM)-1^16^, a ligand that is upregulated on endothelial cells during inflammation. The β_2_ integrin α_L_β_2_ binds intercellular cell adhesion molecule (ICAM)-1, a ligand that is upregulated on various cell types following inflammation. In contrast, α_M_β_2_ is more promiscuous and recognises a range of ligands including ICAM-1 and fibrinogen, which are upregulated at sites of tissue injury and during active coagulation. Integrin-mediated adhesion, via β_1_ and/or β_2_ integrins, is vital to neutrophil activation^17^, leading to the production of reactive oxygen species (ROS) and NETosis^18–21^. Moreover, evidence indicates that integrin inhibition reduces NET release^15,20^.

ARDs are generally characterised by immune dysfunction, with many groups exploring the immune differences in patients with SLE and RA. Given the presence of autoantibodies, it is unsurprising to find that defects in B and T cell regulation have been described in both SLE and RA. B cells contribute to pathology not only through antigen presentation, but also by producing autoantibodies^22^. Studies in RA found that autoantibodies stimulate the production of pro-inflammatory cytokines^22^, which promote T cell, B cell and macrophage activation^22,23^. Greater peripheral B cell activation was observed in patients with SLE^24^, with cells being more sensitive to cytokine stimulation^25^, displaying abnormal receptor-mediated signalling^26^ and having a greater propensity to undergo epitope spreading^27^.

Th1 cells have conventionally been considered to drive RA pathology, however there is growing interest in other T cell subsets. Th17 cells secrete pro-inflammatory mediators that suppress regulatory T cell (Treg) generation^28,29^. Elevated Th17 and reduced Treg differentiation have been described in RA patients, which promote inflammatory cell phenotypes^30^. In addition, Tregs isolated from RA patients have limited suppressive activity^31,32^, which is attributed to low expression of cytotoxic T-lymphocyte-associated protein 4 (CTLA-4)^32^. This reduced Treg population, with defective suppressive capability, fails to suppress autoreactive T cells^33^. Aberrant T cell activation has also been linked to SLE pathology, with reports documenting abnormal TCR signalling and T cell hyper-responsiveness arising through defects in an array of signalling molecules^34–39^.

Macrophages also contribute to RA pathology through pro-inflammatory cytokine production, ROS generation, release of matrix-degrading enzymes, phagocytosis and antigen presentation^40^. Monocytes isolated from SLE patients have elevated expression of activation markers and adhesion molecules^41–44^. Aberrant cytokine production has also been described in SLE-derived monocytes^25,45,46^, which promote the production of anti-dsDNA autoantibodies by B cells^47,48^.

Studies identified neutrophils in the synovial fluid of RA patients^49–51^, with further reports finding increased neutrophil activation^52–54^. Neutrophils have also been implicated in RA mouse models^55–57^. RA neutrophils are prone to spontaneous NETosis, with elevated NETs being reported in RA serum that correlated with anti-citrullinated protein antibodies (ACPA) titres and markers of inflammation^14^. Adaptive immunity has an established role in SLE pathology, however in more recent years, neutrophils are being increasingly studied. Early studies noted that SLE sera induced neutrophil aggregation and interfered with phagocytosis and degranulation^58^. Uptake of circulating nucleosomes activates neutrophils^59^, which leads to the secretion of antibacterial proteins. Elevated bactericidal proteins have been documented in SLE serum, which correlate with autoantibody titres^60^ and disease activity^61–63^. NETosis releases dsDNA and inflammatory cytokines, which also correlate with anti-dsDNA antibodies titres^64^.

The presence of circulating autoantibodies in SLE and RA is well-established, and used to aid diagnosis, predict disease progression and monitor response to treatment. Patients with either SLE or RA commonly present with a multitude of autoantibodies, with a large heterogeneity of antigenic targets^65,66^, each believed to confer pathogenic effects. Early reports found that sub-fractions of SLE-IgG were able to activate cultured endothelial cells and promote adhesion of the monocytic U937 cell line^67^. Some groups have specifically examined the effects of IgG upon neutrophil activation, highlighting an increased propensity of patient-derived neutrophils to undergo spontaneous NETosis as well as serum IgG being able to induce NET release^14,68–70^. However, whilst these studies report IgG effects upon an end-stage functional outcome of neutrophil activation (NETosis), there does not appear to be any evidence examining involvement of β_1_ and β_2_ integrins, which occurs at an early stage of neutrophil activation.

The precise effects of purified ARD-IgG upon neutrophil integrin activation and adhesion are poorly characterised. Given the importance of neutrophil activation to ARD immunopathology, determining the effects of ARD-IgG upon neutrophil function may elucidate the mechanisms through which neutrophils contribute to disease progression. Therefore, we examined the effects of SLE- and RA-IgG on β_1_ and β_2_ integrin-mediated adhesion and activation.

## Results

### Patient demographics

Neutrophils were isolated from 12 healthy controls (HCs), 12 SLE patients and 7 RA patients. IgG was purified from a separate cohort of 9 HCs, 5 SLE patients and 9 RA patients. Demographics, clinical history and treatments of all subjects at the time of sample collection are listed in Table 1. All purified IgG samples tested negative for both PR3- and MPO-anti-neutrophil cytoplasmic antibody (ANCA), which excluded any effects being mediated by these traditional anti-neutrophil antibodies. Only RA-IgG samples tested positive (>5 U/ml) for ACPA. Our SLE cohort displayed presented with a variety of manifestations: 59% presented with renal involvement; 29% had arthritis; 12% had pulmonary involvement and 24% presented with photosensitive rashes. All of our RA cohort presented with articular involvement. In addition, 13% were hypertensive, 13% presented with rheumatoid nodules and 25% had pulmonary involvement. All patient fulfilled relevant classification criteria with stable disease. Patients with active disease requiring medication change at the time of sampling were excluded.

**Table 1:** Patient clinical and demographic data.

	Healthy Control	SLE	RA
Cohort size (n)	21	17	16
Age (years ± SD)	33 ± 8.1	38 ± 6.8	57 ± 9.1
Sex (M:F)	7:14	2:15	4:12
Clinical Features (n)	—	Joint (5), Lung (2), Renal (10), Skin (4)	CVD (2), Joint (16), Lung (4), Skin (2)
Treatments (n)	—	Aspirin (8), HCQ (12), MMF (17), Pred (17), Tacrolimus (3)	Aspirin (1), HCQ (10), Humira (1), MTX (4), Pred (2), RTX (3), SSZ (1)
**IgG Binding Characteristics**			
ACPA (U/ml)	2.16 ± 0.10	2.38 ± 0.34	124.76 ± 106.12
RF (% positive)	0	0	100
ANA (% positive)	0	100	22.2
Anti-dsDNA (% positive)	0	80	0
MPO-ANCA	ns	ns	ns
PR3-ANCA	ns	ns	ns

Clinical details were recorded for all subjects. At the time of blood donation: 2 RA patients had cardiovascular features (hypertension), 16 RA and 5 SLE patients had inflammatory arthritis, 4 RA patients and 2 SLE patients had interstitial lung disease, 2 RA patients had rheumatoid nodules, 4 SLE patients had SLE-related rashes and 10 SLE patients had nephritis. Abbreviations: ACPA, anti-citrullinated protein antibody; ANA, anti-nuclear antibody; ANCA, anti-neutrophil cytoplasmic antibody; CVD, cardiovascular disease; HCQ, hydroxychloroquine; MMF, mycophenolate mofetil; MPO, myeloperoxidase; MTX, methotrexate; ns, not significant; PR3, proteinase 3; Pred, prednisolone; RF, rheumatoid factor; RTX, rituximab; SSZ, sulfasalazine.

### Purified SLE- and RA-IgG both enhance hydrogen peroxide production

**Figure 1:**
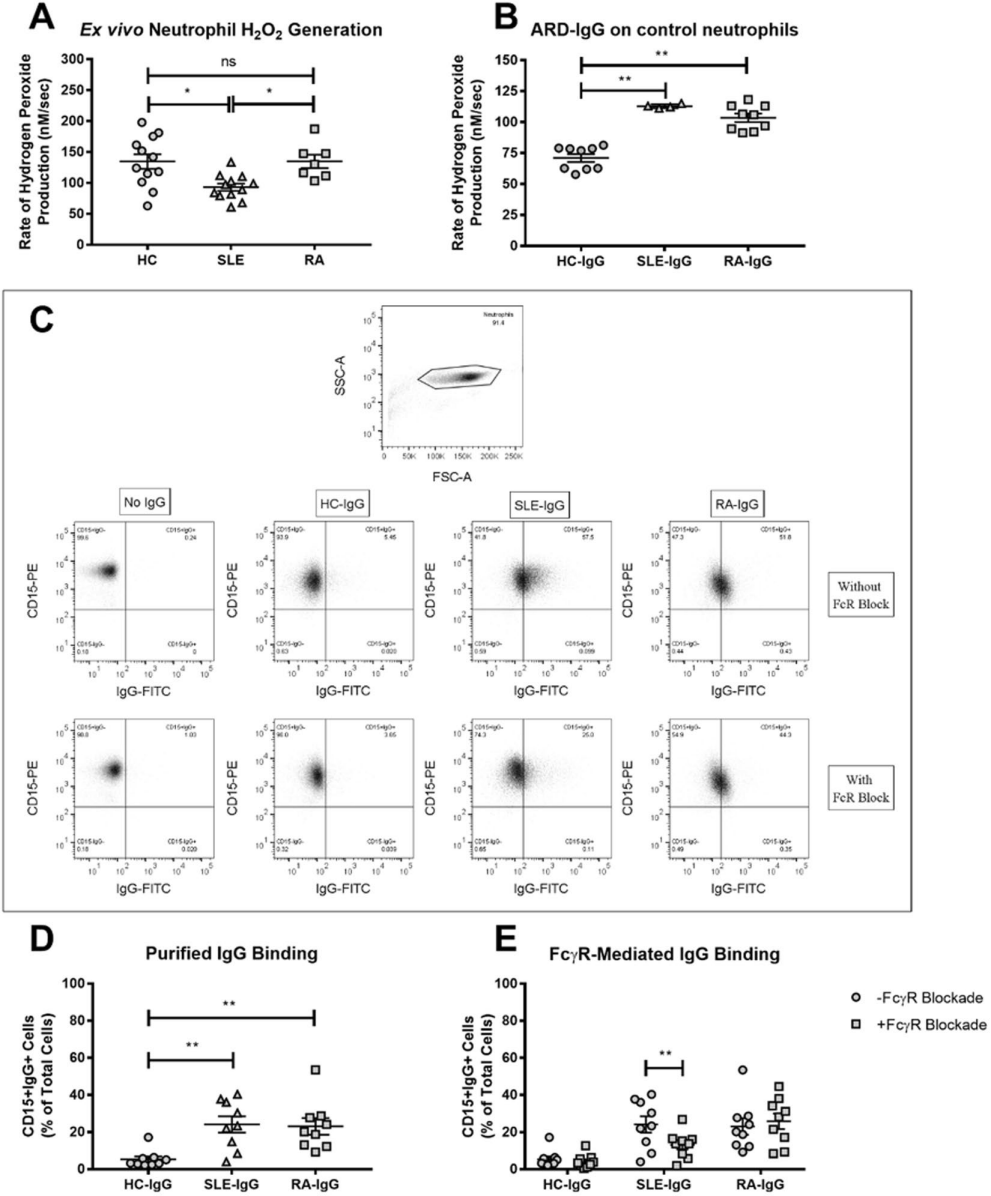
SLE- and RA-IgG both bind neutrophils and prime neutrophils. (**A**) Neutrophils were isolated from patients with SLE (n = 12) or RA (n = 7) and healthy volunteers (n = 12), and rates of H_2_O_2_ production in response to 50 nM PMA using Amplex UltraRed. Data are presented as each point representing a patient sample tested with the mean and SEM also being displayed and were analysed by Kruskal-Wallis test (p = 0.0039) with a Dunn’s multiple comparison test. (**B**) H_2_O_2_ generation in control neutrophils was assessed in the presence of ARD-IgG in the presence of 50 nM PMA. Data are displayed as the mean and SEM from three independent experiments, with each point representing the average of IgG sample over the experimental repeats. Data were analysed by Kruskal-Wallis test (p < 0.0001) with a Dunn’s multiple comparison test. (**C**) Representative flow cytometry plots examining binding for purified IgG to human neutrophils. Isolated neutrophils were incubated with pooled IgG, composed of 5 different IgG samples in the absence or presence of FcγR blockade, and stained for CD15 and human IgG. IgG binding was determined by quantifying the amount of CD15^+^IgG^+^ cell within the neutrophil gate (**D**) IgG binding experiments were performed using neutrophils from 9 different healthy donors, with mean and SEM being displayed. Statistical significance was tested by a Kruskal-Wallis (p = 0.0010) test with a Dunn’s multiple comparison test. (**E**) Additional experiments examined pooled IgG binding in the presence of FcγR blockade and the number of CD15^+^IgG^+^ cells quantified by flow cytometry. Data are presented as the mean and SEM of nine donors (with each point representing a different neutrophil donor), which were compared to the number of CD15^+^IgG^+^ cells previously determined. Data were tested by repeated measures two-way ANOVA a Bonferroni’s multiple comparison test. ns = no significance, *p < 0.05, **p < 0.01.

To examine whether there were intrinsic differences between neutrophils derived from our patient and control populations, we first examined hydrogen peroxide (H_2_O_2_) generation in *ex vivo* SLE-, RA- and HC-derived neutrophils. These experiments demonstrated that SLE-neutrophils had significantly slower rates of H_2_O_2_ production compared to both HC- and RA-neutrophils (Fig. 1A). We also found no significant difference between HC- and RA-neutrophils in our assay. To build on these experiments, we subsequently explored the effects of purified IgG upon neutrophils isolated from healthy volunteers, to determine whether the presence of autoantibodies drive the activated neutrophil phenotype reported among patient populations. Here, we found that pre-conditioning neutrophils with SLE- or RA-IgG induced significantly higher rates of H_2_O_2_ generation (Fig. 1B), indicating that the presence of autoreactive IgG may prime neutrophils to enhance H_2_O_2_ production following stimulation. We therefore found that whilst *ex vivo* SLE-neutrophils displayed slower rates of H_2_O_2_ generation, treatment with ARD-IgG increased H_2_O_2_ production rates in HC-neutrophils.

### ARD-IgG binds neutrophils

To determine whether purified IgG bound neutrophils, we employed a flow cytometry approach. HC neutrophils were incubated with pooled IgG (5 different IgG samples per group), chosen at random from each group, and stained for CD15 and human IgG. Neutrophils were then evaluated by flow cytometry, being selected based on their forward and side scatter properties (Fig. 1C). Gated neutrophils were subsequently analysed based on CD15 and the presence of human IgG, both in the absence and presence of FcR blockade (Fig. 1C). Greater numbers of CD15^+^IgG^+^ cells were observed with both SLE- and RA-IgG compared to HC-IgG (Fig. 1D). Building on this dataset, we also examined IgG binding in the presence of a FcR blocking reagent (Miltenyi, UK), which only reduced binding of SLE-IgG, indicating a contribution from FcγR-mediated interactions with this IgG source (Fig. 1E). Therefore, all subsequent functional studies were performed with FcγR blockade to specifically examine antigen-specific mediated effects.

### ARD-IgG displays differential effects upon neutrophil adhesion and trans-endothelial migration

**Figure 2:**
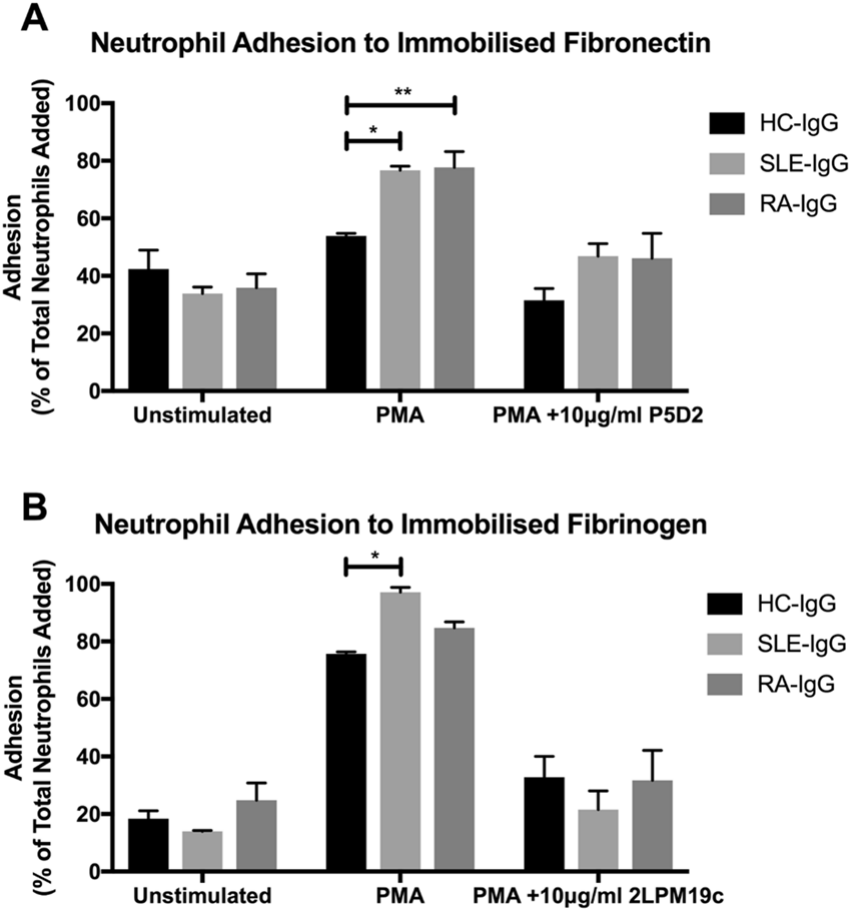
SLE- IgG enhances both neutrophil β_1_ and β_2_ integrin activation. BCECF-AM labelled neutrophil adhesion to immobilised integrin ligands was assessed in the presence of purified IgG to evaluate integrin activation. (**A**) 20 μg/ml fibronectin was immobilised and neutrophil adhesion examined in the absence or presence of 20 nM PMA and the 10 μg/ml pan-β_1_ integrin blocking antibody P5D2. (**B**) 200 μg/ml fibrinogen was immobilised and neutrophil adhesion evaluated in the absence or presence of 20 nM PMA and the 10 μg/ml α_M_β_2_-specifc blocking antibody 2LPM19c. In both cases, data are presented as the mean and SEM from three independent experiments and analysed by two-way ANOVA with a Dunnett’s multiple comparison test. *p < 0.05, **p < 0.01.

To evaluate whether β_1_ or β_2_ integrin-mediated adhesion were modulated by purified IgG, we evaluated neutrophil adhesion to their respective ligands, fibronectin (via β_1_ integrins) and fibrinogen (α_M_β_2_ integrin) in the presence of individual SLE- (n = 5), RA- (n = 7) or HC-IgG (n = 6) samples. In the absence of phorbol 12-myristate 13-acetate (PMA), the effects of SLE- and RA-IgG upon neutrophil adhesion to both fibronectin and fibrinogen were identical to those of HC-IgG (Fig. 2A,B). In contrast, following PMA stimulation, both SLE- and RA-IgG increased neutrophil adhesion to fibronectin (Fig. 2A) compared to HC-IgG. Interestingly, only SLE-IgG significantly increased neutrophil adhesion to immobilised fibrinogen (Fig. 2B). The addition of pan-β_1_ integrin (P5D2) or α_M_β_2_-specific (2LPM19c) blocking antibodies reduced PMA-induced binding to values similar to unstimulated cells (Fig. 2A,B). Thus, PMA-stimulated neutrophil adhesion to fibronectin was enhanced by both SLE and RA-IgG, but to fibrinogen by SLE-IgG only.

**Figure 3:**
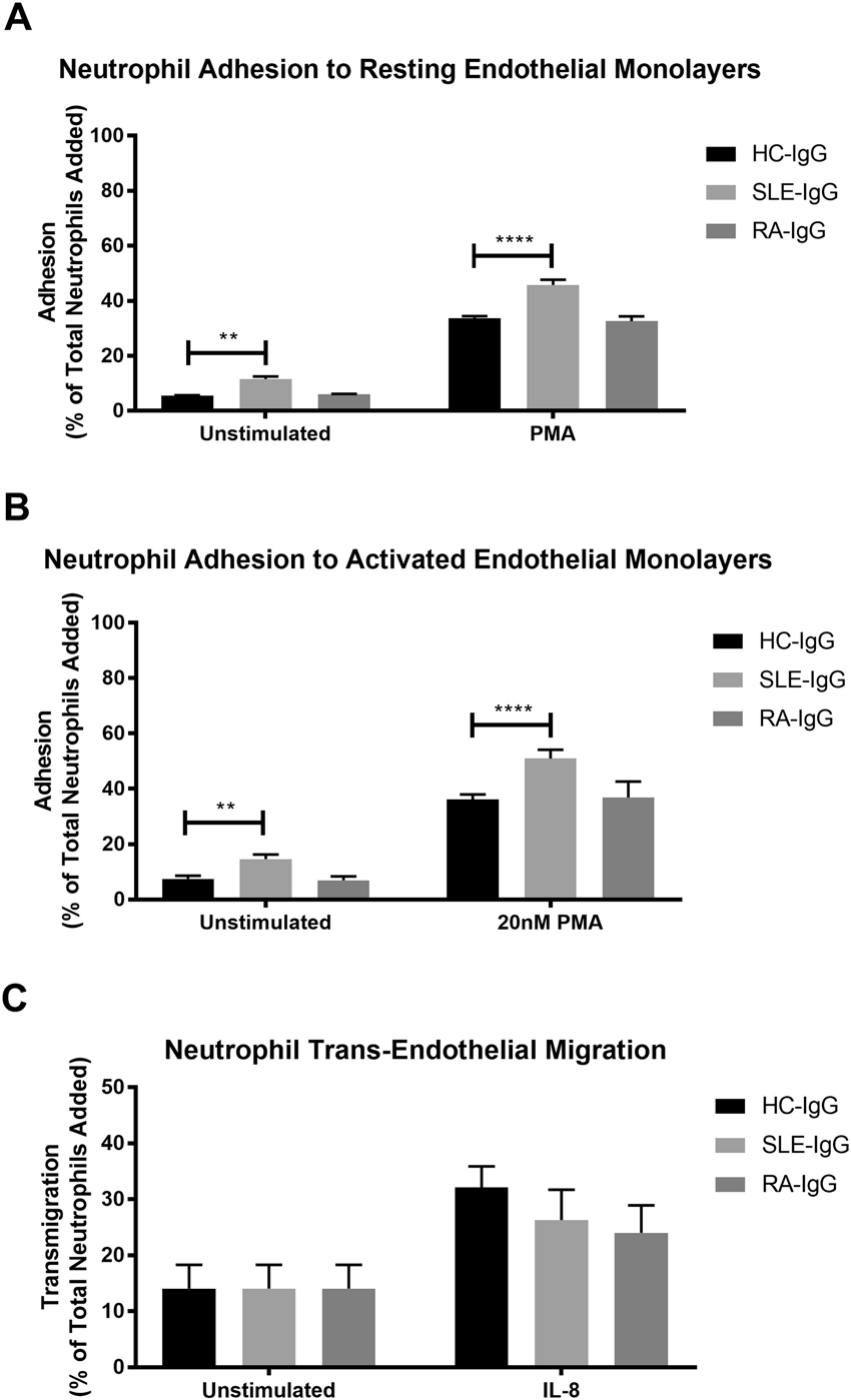
SLE- IgG increased neutrophil adhesion to endothelial cells, but not trans-endothelial migration. BCECF-AM labelled neutrophil adhesion was assessed in the absence or presence of purified IgG and 20 nM PMA to (**A**) resting endothelial cells and (**B**) endothelial cells that had been activated with TNF-α prior to experimentation. (**C**) Fluorescently labelled neutrophil trans-endothelial migration was determined in the absence or presence of purified IgG and 150 ng/ml IL-8, using a trans-well cell culture system and flow cytometry. Cells were quantified by comparing enumerated cells in the lower chamber to the total cells added. In both cases, data are displayed as the mean and SEM of three independent experiments and were tested for significance using two-way ANOVA with a Dunnett’s multiple comparison test. **p < 0.01, ****p < 0.0001.

We then examined the modulatory effects of individual IgG samples upon unstimulated and PMA-induced neutrophil adhesion to human umbilical cord endothelial cells (HUVEC). Interestingly, only SLE-IgG significantly enhanced both unstimulated and PMA-induced neutrophil adhesion to both resting and TNF-α-activated HUVEC compared to control IgG (Fig. 3A,B). In contrast, we found that neither SLE- nor RA-IgG affected basal or chemokine-induced neutrophil trans-endothelial migration compared to HC-IgG (Fig. 3C). Taken together, our data suggest that whilst RA- and SLE-IgG both enhanced PMA stimulated β_1_ integrin-mediated adhesion to fibronectin, only SLE-IgG significantly affected α_M_β_2_-mediated adhesion to fibrinogen as well as cultured HUVEC.

### ARD-IgG increases DNA release by neutrophils

**Figure 4:**
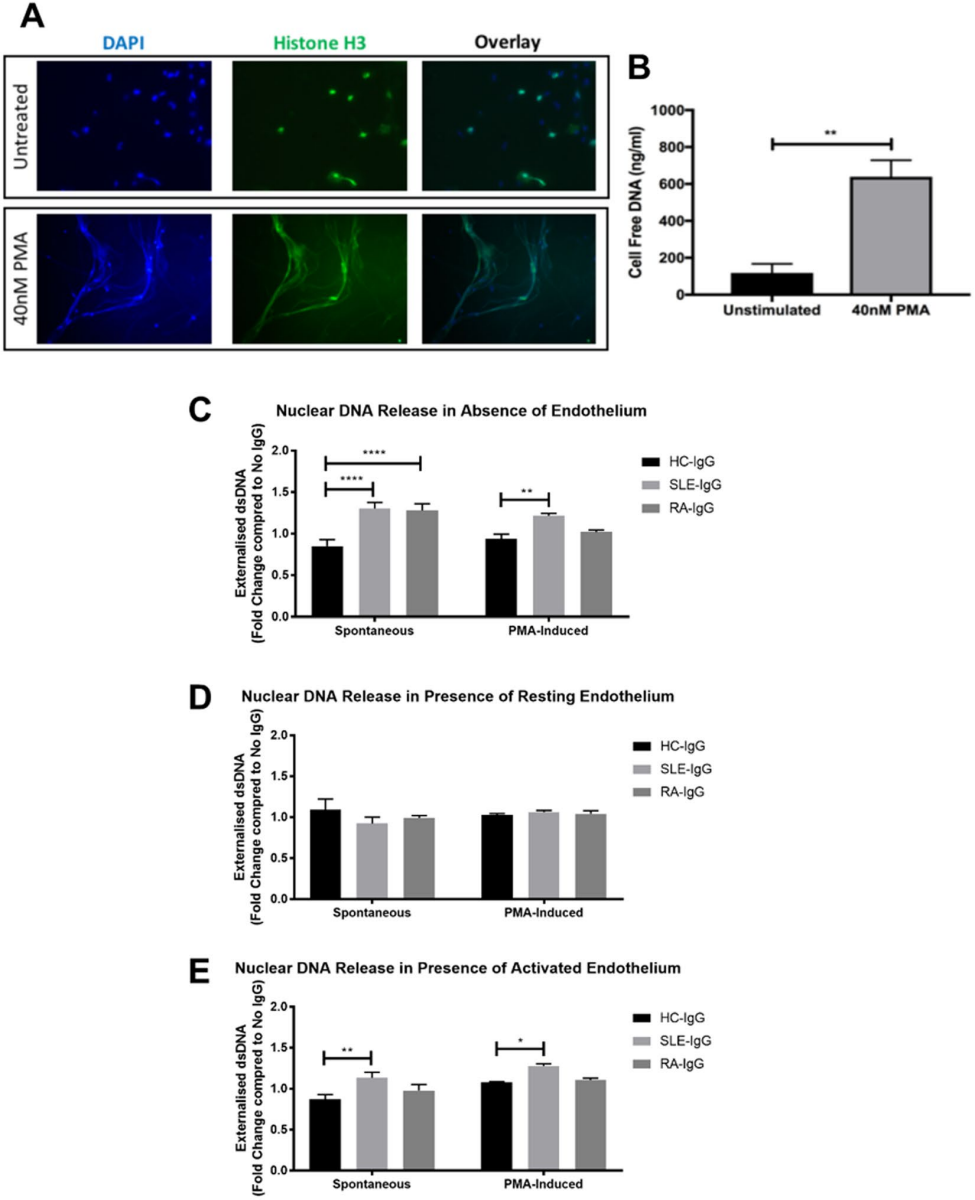
SLE- IgG increased neutrophil NETosis in the presence of endothelial cells. (**A**) PMA-induced NETosis was confirmed by immunofluorescence. Neutrophils were incubated on coverslips in the absence or presence of 40 nM PMA and then fixed. Coverslips were then stained for histone H3 and mounted using a DAPI-mounting medium. (**B**) Cell supernatants from neutrophils cultured in the absence or presence of 40 nM were analysed using the Quanti-iT PicoGreen dsDNA kit. Data are presented as the mean and SEM of three independent experiments. Statistical significance was determined by a Mann-Whitney test. (**C**–**E**) IgG-mediated extracellular DNA release was assessed in (**C**) the absence of an endothelial monolayer; (**D**) the presence of resting endothelial cells; and (**E**) the presence of activated endothelial cells. Neutrophils were cultured for 4 hours in the absence or presence of 40 nM PMA, after which cell supernatants were assessed for cell-free dsDNA. For endothelial cell activation, HUVEC were pre-stimulated with 10 ng/ml TNF-α for 24 hours and washed with warmed PBS prior to addition of neutrophils. Data are presented as the mean and SEM of three independent experiments and analysed using a two-way ANOVA with a Dunnet’s multiple comparison test. *p < 0.05, **p < 0.01, ****p < 0.0001.

We next examined whether IgG could also modulate the release of extracellular DNA, both in the absence (spontaneous) and presence of PMA (inducible). Using immunofluorescence staining for histone H3, we noted that PMA stimulation induced DNA externalisation, characteristic of NET release (Fig. 4A). Comparative analysis of neutrophil supernatants also showed a significant increase in cell-free DNA in PMA-stimulated cells (Fig. 4B). We therefore used measurements of cell-free DNA, to quantitatively measure DNA released by neutrophils, as an indirect measure of NETosis. We then normalised our results against a no IgG control, thus examining any modulatory effects of IgG upon NETosis.

Concurrent with published studies, we also report an increase in spontaneous DNA release by neutrophils in the presence of SLE- or RA-IgG compared to HC-IgG (Fig. 4C). Only SLE-IgG however, significantly elevated PMA-induced DNA externalisation by neutrophils in the absence of endothelial cells (Fig. 4C).

We then studied neutrophil DNA release in co-culture with either steady-state or TNF-α-activated HUVEC to model the cellular interactions that would occur with the vascular endothelium. Interestingly, co-culture of neutrophils with steady-state HUVEC abrogated the increases in both spontaneous and PMA-induced DNA release that we observed with neutrophils stimulated in the absence of endothelial cells (Fig. 4D). However, when HUVEC were activated with TNF-α prior to co-culture, SLE-IgG, but not RA-IgG, significantly increased both spontaneous and PMA-induced DNA externalisation (Fig. 4E).

## Discussion

Abnormalities in neutrophil function contribute to the immunopathology of several ARDs, including SLE and RA. Whilst neutrophils isolated from patients with SLE and RA have been shown to exhibit increased propensities to release NETs, it is not known whether pathogenic autoantibodies directly engage and drive neutrophil dysfunction. We report that whole SLE- and RA-IgG fractions have differential binding to neutrophils *in vitro*, and further demonstrate that ARD-IgG enhance ROS production and DNA release, suggestive of enhanced NETosis. Taken together, these data suggest that ARD-IgG may act as priming agents that enhance neutrophil activation following stimulus.

Many groups have sought to identify the surface molecules implicated in IgG-mediated leukocyte dysfunction. Due to the wealth of potential autoantigens associated with both SLE and RA, identification of a singular antigen target has been met with varying degrees of success and is likely to be completely different in both disease groups. We initially assessed the role of FcγRs, given that immune complexes can interact with neutrophil FcγRs^71^ and induce ROS generation^72^. Indeed, SLE-IgG binding was reduced following FcγR blockade, indicating a degree of FcγR-mediated binding. This reduction may indicate a contribution of FcγR-mediated effects to neutrophil activation in patients with SLE. In our further experiments however, we assessed the antigen-specific effects of purified IgG in the presence of FcγR blockade, we found that both SLE- and RA-IgG were able to enhance β_1_ integrin activation, measured by adhesion to immobilised fibronectin, but only SLE-IgG significantly enhanced α_M_β_2_-mediated adhesion to PMA stimulated neutrophils. These differences in integrin activation may highlight a subtle form of differential regulation by different ARD-IgG upon steady-state and PMA stimulated neutrophils. In this model, it is possible that the presence of ARD-IgG is able to enhance integrin affinity and/or avidity further than that seen with PMA alone. This enhancement may allow for different integrin families to either adopt a high affinity conformation or undergo integrin clustering and therefore allow high avidity interactions with integrin ligands.

Our experiments utilised immobilised fibronectin and fibrinogen, which are found in areas of tissue injury with local activation of coagulation and in regions of vascular endothelial damage where the sub-endothelial basement membrane has been exposed. Our choice of ligands has the advantage of readily allowing us to distinguish the effects of IgG on β_1_-mediated adhesion to ECM/VCAM-1 from the effects on α_M_β_2_-mediated adhesion. Given the wealth of literature on ligand binding motifs, it is reasonable to extrapolate our findings to other integrin ligands for α_M_β_2_ and α_4_β_1,_ such as ICAM-1 and VCAM-1 that play a key role in neutrophil-endothelial interactions. Future work will be aimed at dissecting these ligand preferences further. It is also possible that augmented integrin-mediated binding occurs within the circulation. Fibronectin can be found incorporated into circulating NETs that can promote further neutrophil interactions^73,74^, circulating in plasma, as well as forming a constituent of basement membranes and ECM^75^. In addition, the finding of increased circulating apoptotic endothelial cells in patients with SLE^76^, would provide further endothelial integrin ligands to engage circulating leukocytes within the blood flow.

We found a similar pattern of differential activation with ROS generation, a process requiring α_M_β_2_^77^, that was increased by RA- and SLE-IgG. The requirement of α_M_β_2_, whose affinity is commonly reduced in SLE^78^, may underlie our observation of reduced ROS in *ex vivo* SLE-derived neutrophils. Differences in treatment regimens between groups may also account for the reduced rates of H_2_O_2_ generation. If we consider both the myelosuppressive and immunosuppressive effects of mycophenolate mofetil and prednisolone, as well as their half-lives (17.6 and 2.6 hours respectively)^79,80^, which all SLE patients were prescribed, it is possible that the activity of freshly isolated neutrophils may still be pharmacologically suppressed. Alternatively, reduced H_2_O_2_ production may be a result of metabolic exhaustion arising from systemic activation prior to cell isolation.

Integrin activation is central to leukocyte migration, therefore we examined whether ARD-IgG affected neutrophil adhesion to, and migration across, the endothelium. Interestingly, SLE-IgG, but not RA-IgG, significantly augmented neutrophil adhesion to HUVEC, while migration across an IL-8 gradient was unaffected by ARD-IgG. This observation may suggest that the primary effects of ARD-IgG centre on the firm adhesion stage of leukocyte extravasation, as opposed to enhancing both adhesion and trans-endothelial migration. Interestingly, we also noted differences in unstimulated adhesion, with no significant differences observed to immobilised fibronectin or fibrinogen but increased baseline adhesion to HUVEC in the presence of SLE-IgG. This difference may be explained by the presence of numerous additional adhesion molecules on the endothelial monolayer, which are absent in immobilised integrin ligand. For examples, endothelial cells would also express ICAM-1 and ICAM-2, which are ligands for both α_L_β_2_ and α_M_β_2_ on the neutrophil. These differences highlight the complex interactions occurring between integrins and adhesion molecules under physiological conditions.

Endothelial co-culture modulated the ARD-IgG effects on NETosis. In particular, the presence of a quiescent endothelium abrogated the effects of SLE- and RA-IgG on DNA externalisation observed in the absence of endothelial cells. Stimulation of the endothelium however, restored elevated SLE-IgG induced NETosis, implicating an endothelial contribution. These results may indicate that the presence of ARD-IgG may enhance neutrophil interactions with their external environment and promote neutrophil activation to regions of endothelial activation and dysfunction. A limitation to these experiments is the use of cell-free DNA as an indirect measurement of NETosis. Whilst our approach allowed us to quantitatively compare DNA externalisation to assess the effects upon NETosis, we lose the ability to specifically assess for markers widely accepted to be associated with NET structure, such as citrullinated histones and MPO. The development of a quantitative NET standard to allow the precise measurements of DNA-protein constructs accepted as NETs would resolve this issue.

We administered FcR blockade in our experiments to focus upon the antigen-specific nature of our observations. To further study this interaction additional experiments could be performed using F(ab′)_2_ fragments to ensure cellular effects were mediated solely through antigen-specific binding. Differences in IgG post-translational modifications of the Fc region may also contribute to the physiological effects of IgG. In particular, modifications such as glycosylation and sialylation are believed to mediate cellular effects of IgG^81–85^, and may potentially account for differences between ARD- and HC-IgG. Future experiments however, are required to explore the contribution of any differences in post-translational modifications between IgG groups and were beyond the scope of this study.

**Figure 5:**
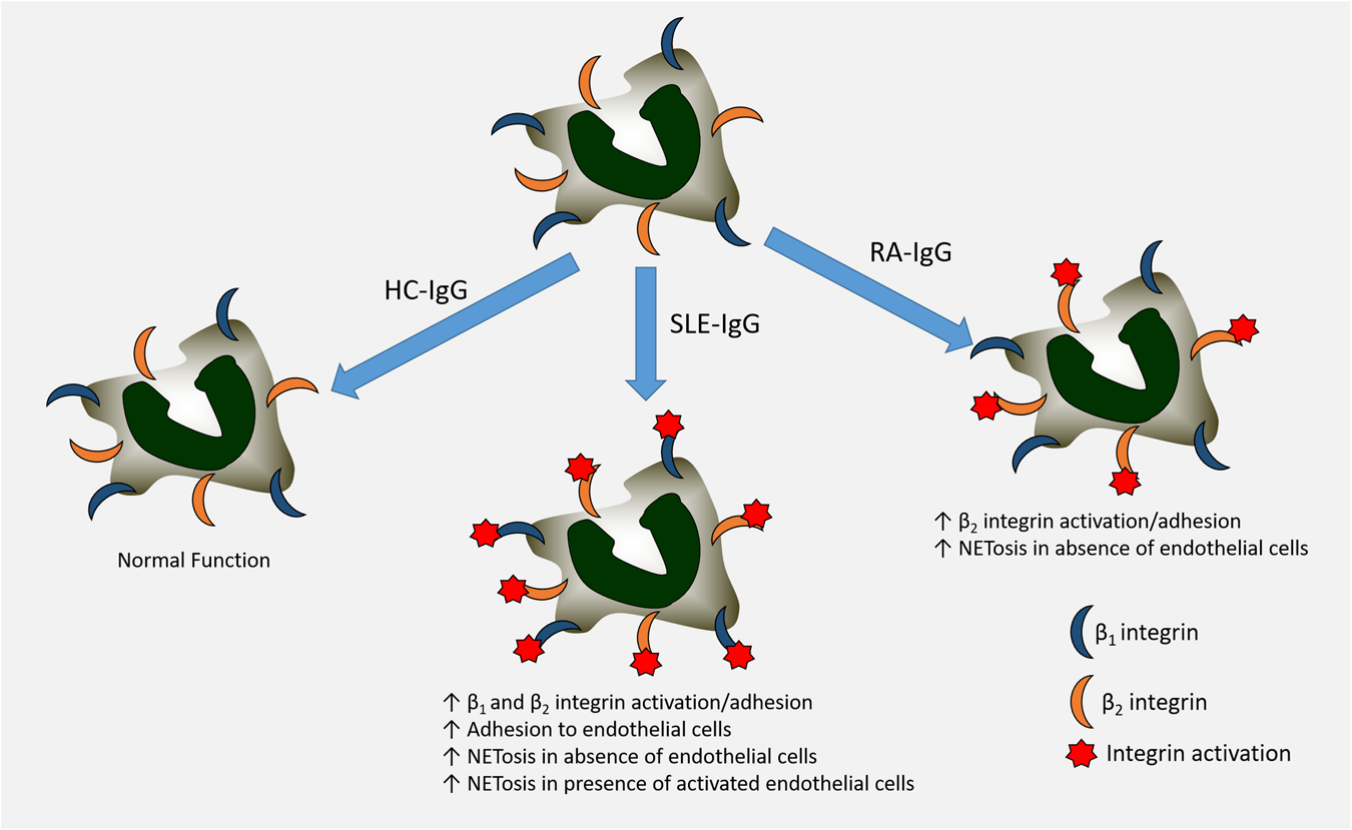
Schematic of overall findings. SLE- and RA-IgG displayed differential effects upon HC-neutrophils in our experiments. SLE-IgG enhanced both β_1_ and β_2_ integrin-mediated adhesion and also adhesion to endothelial cells. In contrast, RA-IgG only increased β_2_ integrin-mediated adhesion. We observed higher levels of NETosis both in absence and presence of activated endothelial cells with the addition of SLE-IgG, whilst RA-IgG only enhanced NETosis in the absence of an endothelial monolayer.

These different patterns of IgG-mediated integrin activation and neutrophil function may help elucidate the differential mechanisms driving vascular damage and dysfunction in these different ARDs. In conclusion, we found a differential pattern of autoantibody binding and integrin activation in steady-state and stimulated neutrophils exposed to purified whole IgG from patients with SLE or RA, which was modulated by the activation state of the endothelium (Fig. 5). Further work is now underway to explore the translational implications of our findings to highlight novel therapeutic targets.

## Materials and Methods

### Patients

Whole blood and serum were obtained by informed consent from 33 patients fulfilling classification criteria; 16 RA^86^ and 17 SLE^87^, as well as 21 HCs. Ethical approval was given by the UK National Research Ethics Committee and NHS Permission was granted for the study to be undertaken at University College London Hospitals NHS Foundation Trust (IRAS ID: 39531; REC Ref: 13/LO/0900; CSP number 39531) and all experimental procedures were performed in accordance with the Declaration of Helsinki and compliant with the principles of Good Clinical Practice.

### IgG purification

Whole IgG was purified by passing patient or HC serum through protein G agarose spin columns (Thermo Scientific, UK) and eluted using 0.1 M glycine (Sigma, UK). Eluted IgG was concentrated, washed and dialysed into endotoxin-free PBS by sequential centrifugations for 20 minutes at 2050 g using centrifugal filter units (Merck Millipore, Ireland). Concentrated IgG was then passed through endotoxin removal columns (Thermo Scientific, UK) and confirmed to be endotoxin-free by EndoLISA (Hyglos, Germany) (<0.06 endotoxin units/ml).

### Autoimmune rheumatic disease-associated IgG binding characterisation

Titres of ACPA in purified IgG samples were quantified using the EDIA anti-CCP-2 kit (Euro Diagnostica, Sweden). Titres of PR3-ANCA and MPO-ANCA in purified IgG were determined using the WIESLAB ANCA screen kit (Euro Diagnostica, Sweden). Quantification of anti-nuclear antibodies (ANA), anti-double stranded DNA antibodies (anti-dsDNA) and rheumatoid factor (RF) were all performed in the University College London Hospital laboratory.

### Neutrophil isolation

Citrated whole blood was obtained by venepuncture and red blood cells removed by dextran sedimentation for 45 minutes. Neutrophils were isolated from the cell-enriched plasma by Percoll PLUS (Sigma, UK) gradient centrifugation at 700 g for 30 minutes and resuspended in phenol-free RPMI (Thermo Scientific, UK) supplemented with 10% FCS (Thermo Scientific, UK) and 2mM L-glutamine (Lonza, Switzerland). Isolated neutrophils were counted using a 0.4% Trypan Blue solution and diluted to 2 × 10^6^ neutrophils/ml to ensure the same number of viable cells were used between groups and experiments. For experiments examining the effects of IgG, cells were treated with a FcR blocking reagent (Miltenyi, UK) (2 μl per 1 × 10^6^ neutrophils) for 20 minutes prior to addition of 200 μg/ml purified IgG.

### Endothelial cell culture

HUVEC at passage 2 (P2) (Lonza, Switzerland) were cultured in endothelial growth media 2 (Lonza, Switzerland) with 10% FCS (Thermo Scientific, UK) and 2mM L-glutamine (Lonza, Switzerland) to P5 for all experimental procedures. For endothelial activation, HUVEC were treated with 10 ng/ml TNF-α (R&D Systems, UK) for 24 hours and then washed with warmed HBSS prior to experimentation.

### IgG binding

Neutrophils were cultured with 200 μg/ml IgG for 2 hours, fixed in 2% paraformaldehyde (PFA) (Sigma, UK) for 15 minutes, and stained using PE-conjugated anti-CD15 (clone W6D3) and FITC-conjugated anti-human IgG (clone G18-145) (BD Biosciences, UK) for 30 minutes at room temperature in the dark. Stained cells were washed twice in a sodium HEPES buffer (20 mM HEPES, 140 mM NaCl, 2 mg/ml glucose, 0.3% BSA) and analysed using a FACSVerse (BD Biosciences). Resulting data was analysed using FlowJo (TreeStar Inc., UK). IgG binding was calculated as the percentage of CD15^+^ neutrophils that were also positive for human IgG.

### Neutrophil adhesion

Fibronectin (Sigma, UK) (for β_1_ integrin engagement) and fibrinogen (Sigma, UK) (for α_M_β_2_ integrin engagement) were immobilized in 96-well black MaxiSorp microplates (Thermo Scientific, UK) and blocked with 2% fish-skin gelatin (Sigma, UK), to reduce non-specific neutrophil binding (Fig. S1). HUVEC were cultured in 96-well black tissue culture plates (Thermo Scientific, UK). HC-neutrophils were labelled with 2.5 μM 2′,7′-bis-(2-carboxyethyl)-5-(and-6)-carboxyfluoresceinacetoxymethyl ester (BCECF-AM) (Life Technologies, UK) for 30 minutes at 37 °C. Stained cells were washed twice in the sodium HEPES buffer by centrifugation at 300 g for 5 minutes and resuspended to 1 × 10^7^ neutrophils/ml. 5 × 10^5^ neutrophils were added to wells containing 20 nM PMA and 200 μg/ml IgG for 30 minutes at 37 °C. Fluorescence was measured using a Tecan GENios Spectra FLUOR plate reader (Tecan UK Ltd., UK). Plates were washed and read again. Adhesion was calculated by comparing the fluorescence of washed wells to initial fluorescence.

### Neutrophil trans-endothelial migration

HUVEC were grown to confluence on transwell inserts (Millipore, UK). HC-neutrophils were isolated, washed with M199 media (Invitrogen, UK) labelled with a 0.5 µM CellTracker (Invitrogen, UK). Labelled cells were washed with M199 media by centrifugation at 300 g for 5 minutes and resuspended to 5.5 × 10^6^ neutrophils/ml in M199 media supplemented with 1% FCS. Transwells were washed with M199 and 1.1 × 10^6^ neutrophils added to the upper chamber. Transmigration was assessed both in the absence or presence of 150 ng/ml IL-8 in the lower chamber for 90 minutes. Migration was evaluated by flow cytometry, using CountBright absolute counting beads (Invitrogen, UK) to enumerate the number of cells in the lower chamber. Percent transmigration was calculated by comparing the number of cells in the lower chamber and the number of neutrophils added to the upper chamber.

### Hydrogen peroxide generation

2 × 10^6^ HC-neutrophils were incubated with 200 μg/ml IgG for 1 hour at 37 °C before addition of 0.5U/ml HRP (Sigma, UK) and 60 nM Amplex UltraRed (Invitrogen, UK). 4 × 10^5^ cells were added to each test well of a 96-well black microplate (Thermo Scientific, UK) and fluorescence measured by FLUOstar Omega microplate reader (BMG Labetech, Germany). H_2_O_2_ generation in response to 50 nM PMA was recorded over time and rates (expressed in nM/sec) determined using Omega Mars Analysis software (BMG Labtech, Germany). In separate experiments, H_2_O_2_ generation was assessed in patient- and HC-derived neutrophils, however in the absence of autologous IgG.

### Immunofluorescence staining for neutrophil extracellular traps

Glass coverslips (Fischer, UK) were sterilized with and coated with 200 μg/ml fibrinogen (Sigma, UK) at 4 °C. 5 × 10^5^ neutrophils were then added to coverslips and stimulated for 4 hours, after which they were fixed for 15 minutes with 4% PFA at room temperature. Coverslips were blocked in a blocking solution (10% goat serum/1% BSA/2 mM EDTA/HBSS/0.1% Tween-2) overnight at 4 °C, then incubated with 1 µg/ml anti-histone H3 antibody (ab1791, Abcam, UK) and then 2 µg/ml Alexa Fluor 488-conjugated goat anti-rabbit IgG secondary antibody (Life Technologies, UK), which were diluted in blocking solution, for one hour each at room temperature. After incubations, coverslips were washed with HBSS twice, mounted and sealed on microscope slides with ProLong Gold antifade mountant with DAPI (Invitrogen, UK). Slides were subsequently visualized using a Zeiss Axio Imager.A1 inverted fluorescence microscope (Zeiss, Germany) and images analysed using Image J.

### Extracellular DNA quantification

HC-neutrophils were pre-treated with IgG for 1 hour, after which NETosis was induced by stimulation with 40 nM PMA for 4 hours. Neutrophil supernatants were obtained by centrifugation at 300 g for 5 minutes and then assessed for extracellular DNA using the Quanti-iT PicoGreen dsDNA kit (Invitrogen, UK). Results were then normalised to a no IgG control, thus examining any modulatory effects of IgG.

### Statistical analysis

Data were evaluated using GraphPad Prism. Data were first tested for normality using a Kolmogorov-Smirnov test. In experimental data sets only comparing two groups, a Mann-Whitney test was performed. Where multiple groups were being compared, a Kruskal-Wallis test with a Dunn’s multiple comparison test was used. In data sets with two variables, data were assessed by two-way ANOVA with a Dunnet’s multiple comparison test. A p value below 0.05 was considered significant.

## Supplementary information

Supplementary Figure 1: Fish-skin gelatin reduced non-specific neutrophil adhesion
