# Supplementary material for: Autoimmune rheumatic disease IgG has differential effects upon neutrophil integrin activation that is modulated by the endothelium
Source: Sci Rep. 2019 Feb 4;9:1283. doi: 10.1038/s41598-018-37852-5 (PMC6361939; doi:10.1038/s41598-018-37852-5)
Supplement: Supplementary file 1 — Supplementary Figure 1: Fish-skin gelatin reduced non-specific neutrophil adhesion [file 41598_2018_37852_MOESM1_ESM.docx]

**Autoimmune rheumatic disease IgG has differential effects upon neutrophil integrin activation that is modulated by the endothelium**

Akif A. Khawaja^1,2^, Charis Pericleous^3^, Vera M. Ripoll^2^, Joanna C. Porter^1^†, Ian P. Giles*†^2^

^1^Centre for Inflammation and Tissue Repair, University College London, London, United Kingdom; ^2^Centre for Rheumatology, University College London, London, United Kingdom; ^3^National Heart and Lung Institute, Imperial College London, London, United Kingdom.

†These authors contributed equally to this work.

*Corresponding author: I.P. Giles, Room 411, Rayne Institute, 5 University Street, London, WC1E 6JF, United Kingdom. Tel no: 020 3108 2156, Fax no: 020 3108 2152, Email: [i.giles@ucl.ac.uk](mailto:i.giles@ucl.ac.uk).

**
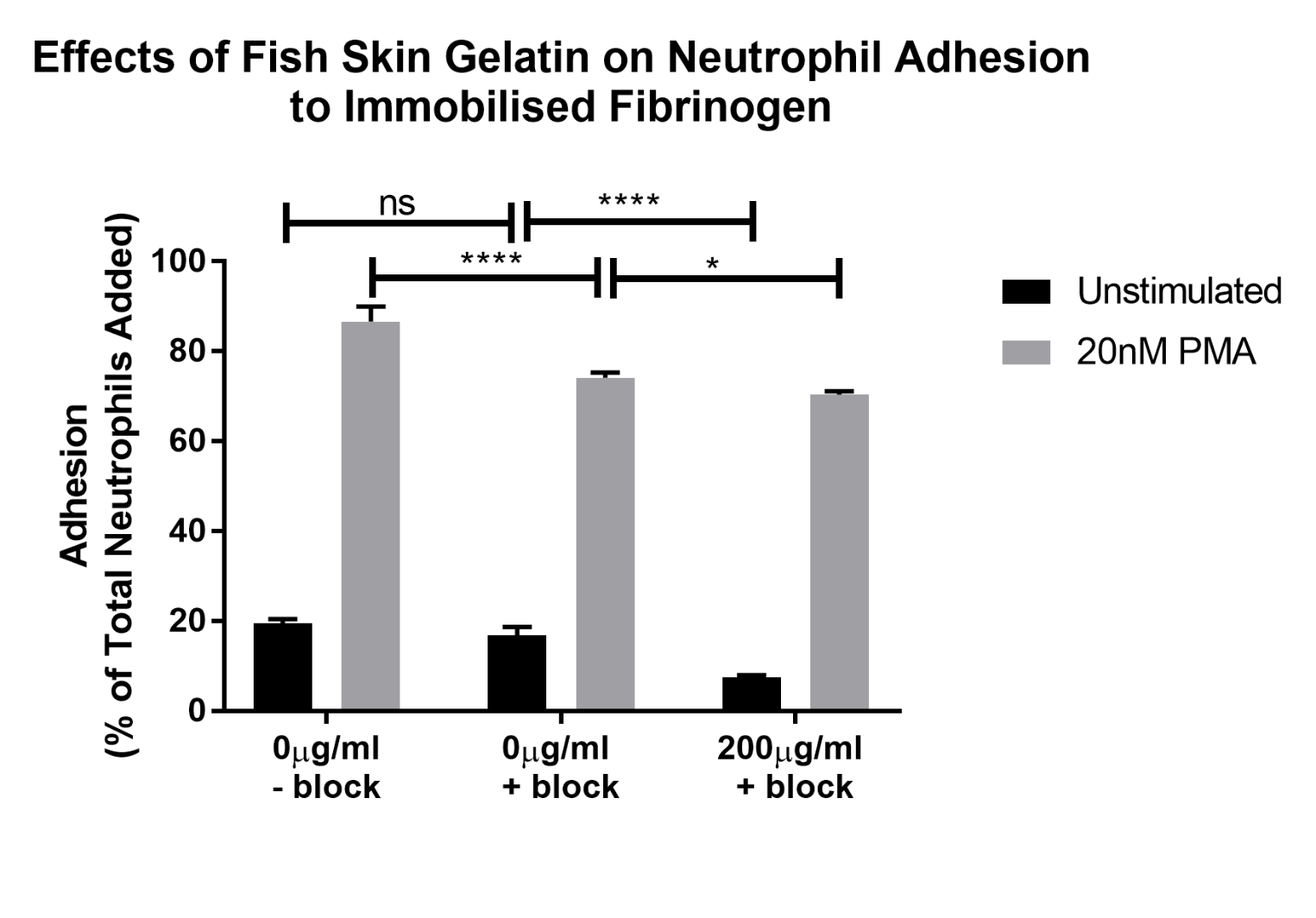
Supplementary Figure 1**

**Supplementary Figure 1: Fish-skin gelatin reduced non-specific neutrophil adhesion**

To verify whether neutrophils would bind the 2% fish skin gelatin blocking reagent, we examined neutrophil adhesion in the absence or presence of the blocking step. BCECF-AM labelled neutrophils were incubated for 30 minutes in wells that had been either left uncoated or had been coated with 200µg/ml fibrinogen. Some uncoated wells were also left unblocked, with the remaining uncoated and 200µg/ml fibrinogen wells being blocked with 2% fish-skin gelatin as described. We noted a significant reduction in the PMA-stimulated neutrophil adhesion in uncoated wells that had been blocked compared to those that had not. The introduction of immobilised fibrinogen further reduced both unstimulated and PMA-stimulated neutrophil adhesion. Data are presented as the mean and SEM of three independent experiments and analysed using a two-way ANOVA with a Dunnet’s multiple comparison test. ns= no significance, *= p<0.05, , ****= p<0.0001.
